# Plasma untargeted metabolomics reveals promising diagnostic metabolites for adrenocortical carcinoma

**DOI:** 10.3389/fphar.2026.1817514

**Published:** 2026-08-21

**Authors:** Yihui Yang, Shuping Shi, Jianqiang Wu, Tingting Xu, Junmei Shang, Jiaxin Yu, Bo Zhang, Xin Liu

**Affiliations:** 1 Department of Pharmacy, Peking Union Medical College Hospital, Chinese Academy of Medical Sciences and Peking Union Medical College, Beijing, China; 2 State Key Laboratory of Complex Severe and Rare Diseases, Peking Union Medical College Hospital, Beijing, China; 3 Center for Biomarker Discovery and Validation, National Infrastructure for Translational Medicine, Institute of Clinical Medicine, Peking Union Medical College Hospital, Chinese Academy of Medical Sciences and Peking Union Medical College, Beijing, China

**Keywords:** adrenocortical carcinoma, diagnostic biomarker, glycocholic acid, plasma metabolites, plasma untargeted metabolomics

## Abstract

Adrenocortical carcinoma (ACC) is a rare and aggressive malignancy with poor prognosis. Surgery is the only curative option for ACC, but recurrence remains high. Effective systemic therapies are still lacking in ACC. Diagnosis currently relies on integrated hormonal, imaging, and histopathological assessments. However, distinguishing ACC from benign adrenocortical adenoma (ACA) remains challenging. To address this, we performed untargeted plasma metabolomics on patients with ACC and ACA, as well as on healthy controls, to identify differential metabolites and elucidate the enriched Kyoto Encyclopedia of Genes and Genomes (KEGG) pathways. Notably, glycocholic acid emerged as a significantly upregulated metabolite in ACC with superior diagnostic accuracy. Furthermore, a metabolic model incorporating six plasma metabolites, including glycocholic acid, distinguished ACC from ACA and healthy controls with an area under the curve (AUC) of 0.9266. These findings demonstrate that plasma metabolomics might serve as a promising tool for the detection of ACC.

## 1 Introduction

Adrenocortical carcinoma (ACC) is a rare and aggressive endocrine malignancy originating from the adrenal cortex, with an incidence of approximately 1–2 per million per year in the general population (Fassnacht et al., 2025). ACC exhibits a bimodal age distribution, occurring most frequently in children younger than 5 years and adults aged 40–50 years and is more common in female individuals than in male individuals (Fassnacht et al., 2025; Flauto et al., 2025; Ilanchezhian et al., 2022). The 5-year survival rate for ACC is generally poor but varies widely by the disease stage, ranging from 18% to 82% (Sarrafan-Chaharsoughi et al., 2025). At present, complete surgical resection is the only potentially curative treatment option for ACC, but the cumulative recurrence rate after R0 resection remains as high as 30%–75% (Fassnacht et al., 2010; Mirallié et al., 2019). Due to the lack of distinct and specific alarm symptoms, the majority of ACC patients are diagnosed at an advanced stage (Crona and Beuschlein, 2019). For unresectable or metastatic cases, mitotane is the only drug formally approved, but the first-line treatment EDP-M (etoposide, doxorubicin, cisplatin, and mitotane) only achieved poor responses in patients (Ghosh et al., 2023; Brönimann et al., 2023). Thus, early diagnosis is pivotal for improving the outcomes in ACC patients.

In clinical settings, ACC accounts for approximately 8%–11% of adrenal tumors, and the remaining subtypes include adrenocortical adenomas (ACA) (80%), pheochromocytomas (7%–10%), and metastases from other tumors (5%–7%) (Libé and Huillard, 2023). Hormonal and imaging investigations are two fundamental assessments for the diagnosis of ACC (Fassnacht et al., 2018). As misdiagnosis is relatively common in ACC, pathological diagnosis is also recommended for suspected ACC. The Weiss scoring system and immunohistochemical staining for SF-1 and Ki-67 are the most commonly used pathological assessment factors (Relav et al., 2023; Tissier et al., 2012; Mete et al., 2022). Comprehensive clinical, biochemical, radiological, and histological assessments are necessary to differentiate ACC from benign adenomas, metastases to the adrenal gland, and pheochromocytomas. Although multiple diagnostic procedures have been proposed, the diagnosis of ACC remains a great challenge because of the rarity and variable clinical features of the disease (Chukkalore et al., 2024). Hence, it is urgent to discover novel biomarkers with remarkable diagnostic and prognostic potential in ACC.

In recent years, steroid metabolome profiling has been widely investigated to discover potential biomarkers for the diagnosis of ACC. Arlt et al. (2011) first applied GC–MS to analyze urine steroid hormone profiles and identified tetrahydro-11-deoxycortisol (THS) as the most discriminative steroid for distinguishing ACC from ACA. Schweitzer et al. (2019) used liquid chromatography–tandem mass spectrometry (LC–MS/MS) to analyze the plasma steroid hormone profiling of ACC and ACA patients and identified six types of steroid hormones, which were significantly increased in ACC compared with ACA and could compose a reliable diagnostic signature for the diagnosis of ACC. Moreover, urine steroid metabolomics can also be used to detect the recurrence of ACC (Chortis et al., 2020). Although most studies have focused on the steroid metabolome in ACC, the metabolic abnormalities based on a broader spectrum of small-molecule metabolites remain unexplored.

In the present study, untargeted metabolomics was performed to assess plasma metabolomic profiles in ACC and ACA patients, as well as in healthy controls. Differential metabolites were identified in the progression of ACC, and the Kyoto Encyclopedia of Genes and Genomes (KEGG) pathway was enriched. Moreover, glycocholic acid was identified as the most significantly upregulated plasma metabolite with the highest diagnostic accuracy in discriminating ACC patients from ACA and healthy controls. We also constructed a metabolic diagnostic model that accurately predicted the diagnosis of ACC patients with an AUC of 0.9266. Our research demonstrated that plasma metabolomics might be a promising tool for the discovery of potential diagnostic markers for ACC diagnosis.

## 2 Materials and methods

### 2.1 Participants and sample collection

The research was approved by the Ethics Committee of the Peking Union Medical College Hospital, Chinese Academy of Medical Sciences, and was conducted according to the principles of the Declaration of Helsinki. All patients provided written informed consent. The study samples were derived from a consecutive clinical case database established at our center from January 2023 to December 2024. Two independent researchers performed the medical record review and data extraction. The inclusion criteria were as follows: (1) patients with ACC or ACA aged more than 18 years; (2) patients with ACC or ACA diagnosed based on pathological findings. The exclusion criteria were as follows: (1) patients who had undergone surgery; (2) patients who were treated with mitotane, ketoconazole, or mifepristone. A total of 152 participants were enrolled, comprising 42 patients with ACC, 56 patients with ACA, and 55 healthy controls. Venous blood samples from patients and healthy volunteers were collected, anticoagulated with EDTA, and centrifuged at 4 °C and 5,000 rpm for 10 min within 30 min of blood isolation. The supernatant was separated, quickly frozen in liquid nitrogen, and stored at −80 °C.

### 2.2 Sample collection

The EDTA plasma samples were thawed on ice. Then, 50 μL of the sample was transferred to a 1.5 mL tube, followed by the addition of 150 μL of ultrapure water, and the mixture was vortexed for 30 s. Subsequently, 400 μL of acetonitrile was added, and the mixture was vortexed for 30 s. The sample was then centrifuged at 14,000 *g* for 10 min, and the supernatant was transferred to a 10 mL glass tube and evaporated to dryness using a rotary evaporator at room temperature. The residue was dissolved in 200 μL of 2% acetonitrile and filtered using an ultrafiltration centrifugal filter with a molecular weight cutoff of 10 kDa. The filtrate was collected and analyzed using LC–MS/MS.

### 2.3 LC–MS/MS analysis

Liquid-chromatographic separation of processed plasma samples was accomplished using a Waters HSS C18 column (3.0 × 100 mm, 1.7 μm) maintained at 45 °C. The mobile phase consisted of solvent A (water containing 0.1% formic acid) and solvent B (acetonitrile). The gradient elution procedure was set as follows: 0–1 min, 2% solvent B; 1–3 min, 2%–15% solvent B; 3–6 min, 15%–50% solvent B; 6–9 min, 50%–95% solvent B; 9–9.1 min, 95%–100% solvent B; 9.1–12 min, 100% solvent B; 12–12.1 min, 100%–2% solvent B; and 12.1–17 min, 2% solvent B. The flow rate was 0.5 mL/min, the column temperature was maintained at 45 °C, the injection volume was 5 μL, and the autosampler temperature was set at 4 °C. Mass spectrometry was performed using a Nexera X2 system (Shimadzu, Kyoto, Japan) coupled with a Triple TOF 5600 quadrupole–time-off-flight mass spectrometer (AB SCIEX, Framingham, Massachusetts, United States). MS/MS spectra were acquired using a QTRAP 6500+ mass spectrometer (Sciex, Framingham, MA) equipped with an electrospray ionization (ESI) source operating in the negative ion mode. The top 10 most abundant precursor ions were selected for MS/MS fragmentation, with a collision energy of 35 ± 15 eV. The temperature of the sample chamber was maintained at 7 °C. Despite the potential suppression of ionization efficiency under negative ion mode, 0.1% formic acid was included in the mobile phase in our experiment as it afforded the best peak symmetry and resolution for the target acidic metabolites while concurrently minimizing background noise and sodium adduct formation. The resulting sensitivity was verified to be adequate for all analytes of interest. Metabolite identification was performed according to the Metabolomics Standards Initiative (MSI) guidelines. In brief, metabolites were annotated based on the following criteria: (1) mass accuracy: the mass tolerance for precursor ions (MS1) was set at ±5 ppm, and for fragment ions (MS2), it was set at ±10 ppm, based on external calibration and lock-mass correction; (2) retention time (RT) matching: retention times were compared with authentic standards when available or with library entries under identical chromatographic conditions (RT tolerance ±0.2 min); (3) MS/MS spectral matching: MS/MS spectra were matched against publicly available databases, including HMDB (version 5.0), METLIN, and mzCloud (search score threshold ≥70%); and (4) confidence levels: metabolite annotations were assigned confidence levels according to MSI guidelines, including Level 1 (confirmed by authentic standard), Level 2 (putative annotation based on library matching), and Level 3 (tentative candidate). In this study, glycocholic acid and the other five metabolites in the diagnostic model were annotated at Level 2 (matched with MS/MS spectra from at least two databases), and all 488 reported metabolites were annotated at Level 2 or Level 3.

### 2.4 Quality control and batch effect correction

In the untargeted metabolomics workflow, a pooled quality control (QC) sample was prepared by mixing equal aliquots of all plasma samples included in this study to serve as a representative reference for system conditioning and drift correction. Prior to LC–MS analysis, all samples were randomized, and the analytical sequence was divided into several batches, each containing a balanced representation of case and control samples. Within each batch, analysis began with eight conditioning QC injections to equilibrate the column, followed by intermittent QC injections after every 10 study samples, while blank samples were periodically analyzed to monitor carryover and contamination. To monitor instrument drift, the relative standard deviation (RSD) of metabolite intensities across QC samples was calculated, and metabolites with QC RSD exceeding 30% were excluded from further analysis. For batch effect correction, we applied the QC-robust spline correction (QC-RSC) method to adjust for signal intensity drift over time, using the QC samples as anchors for correction. The effectiveness of the correction was verified using principal component analysis (PCA), which demonstrated tight clustering of QC samples and a significantly reduced coefficient of variation (CV), confirming robust data quality.

### 2.5 Data analysis and statistics

The identification strategy of the differential metabolites is based on the previously reported method (Liu et al., 2018; Wang et al., 2024). The raw data files were processed using Progenesis QI software (Waters, Milford, MA, United States), including peak alignment, peak picking, peak grouping, deconvolution, and normalization by total compounds. The feature file that was exported was then imported into MetaboAnalyst (http://www.metaboanalyst.ca) for additional analysis. Any variables that were absent in more than 50% of the samples were removed from further statistical analysis, and missing values were estimated using the KNN method. The orthogonal partial least squares discriminant analysis (OPLS-DA) was applied to visualize the differences among the three groups for comparative analysis of their metabolomic profiles. Based on the LC–MS/MS results, software analysis was conducted to plot the comparison of levels in metabolites in plasma between patients with ACC, ACA, and healthy controls and to generate a receiver operating characteristic (ROC) curve for diagnosing adrenocortical carcinoma using plasma levels. Logistic regression was performed to evaluate the diagnostic value of six metabolites, namely, 3-oxododecanoic acid, 5-hydroxy-L-tryptophan, 12-hydroxydodecanoic acid, deoxycholic acid glycine conjugate, D-maltose, and glycocholic Acid, for distinguishing ACC patients from non-ACC controls. Gene Expression Profiling Interactive Analysis (GEPIA, http://gepia.cancer-pku.cn/) was used to analyze the expression of TGR5 in ACC. To evaluate model generalizability and guard against overfitting, 10-fold stratified cross-validation was performed using Python. P-values, false discovery rates (FDRs), and variable importance in projection (VIP) scores were used to compare the differences in detected plasma metabolites among ACC, ACA, and healthy control groups. A two-sided *P*-value of ≤0.05 was considered statistically significant.

## 3 Results

### 3.1 Characteristics of the participants and data quality assessment

**FIGURE 1:**
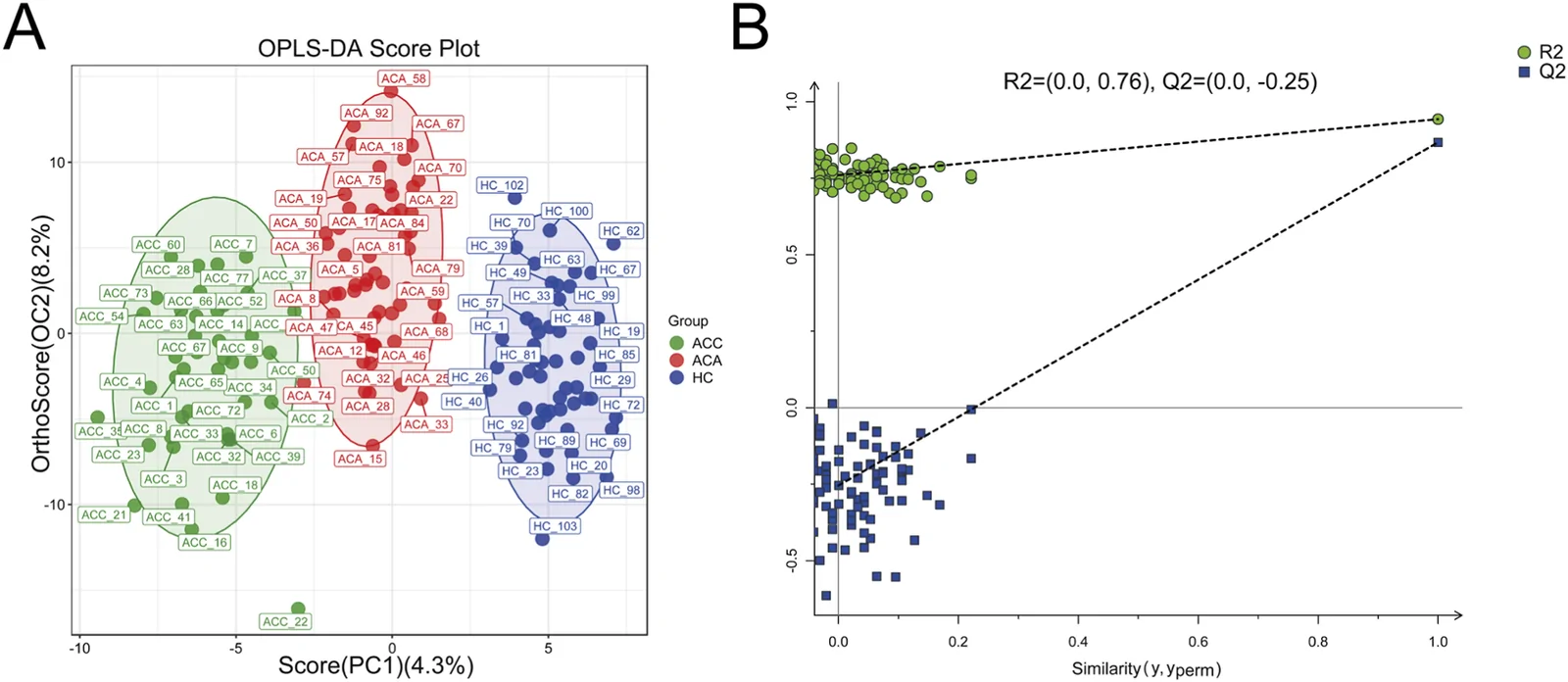
OPLS-DA of the plasma metabolome of ACC patients (n = 42), ACA patients (n = 56), and healthy controls (HCs) (n = 55). **(A)** The OPLS-DA score plot of three groups. **(B)** Permutation test results demonstrated the reliability of the results of OPLS-DA.

In the present study, 153 plasma samples were collected, comprising 42 patients with ACC, 56 patients with ACA, and 55 healthy individuals. The detailed information of the discovery cohorts is shown in Table 1. Untargeted metabolomics was performed using LC–MS/MS to detect the plasma metabolites, and 488 metabolites were identified in three groups. For metabolomics data preprocessing, missing or nonpositive values in the metabolite abundance matrix were imputed using half of the minimum positive value for each metabolite. The imputed data were normalized by total quantified feature abundance to the median sample total, followed by log2 (x + 1) transformation. Pareto scaling was then applied before multivariate statistical analysis. QC samples were included to evaluate analytical stability. Injection-level QC assessment was performed based on the missing/nonpositive feature rate and total quantified feature abundance. PCA was subsequently performed using the processed data to assess QC clustering and overall data stability (Supplementary Figure S1). To investigate the metabolic pattern of three groups, the OPLS-DA was performed, and the results indicated an obvious separation between the three groups, and the plasma metabolome of ACC patients was significantly discriminated from that of the ACA group and the healthy control group (Figure 1A) (Worley and Powers, 2016). Permutation test results demonstrated high predictability and reliability of OPLS-DA, with R^2^Y values of 0.976 and Q^2^ values of 0.889 (Figure 1B).

**TABLE 1:** Characteristics of the discovery cohorts.

Characteristic	Stages	ACC	ACA	HC
Sex, male	​	17/42	27/56	26/55
Age, years, median (range)	​	46.9 (28–72)	49.0 (27–72)	46.9 (26–71)
Hormone active (yes)	​	30 (71.4%)	38 (67.9%)	​
ENSAT stage at initial diagnosis	​	​	​	​
​	I	13 (30.1%)	​	​
​	II	16 (38.1%)	​	​
​	III	5 (11.9%)	​	​
​	IV	8 (19.0%)	​	​
Tumor present at sampling	​	​	​	​
​	Primary tumor	5 (11.9%)	​	​
​	Primary tumor + metastases	6 (14.3%)	​	​
​	Local recurrence	6 (14.3%)	​	​
​	Distant recurrence	10 (23.8%)	​	​
​	Local + distant recurrence	15(35.7%)	​	​

### 3.2 Alteration of plasma metabolites in stages of ACC progression

**FIGURE 2:**
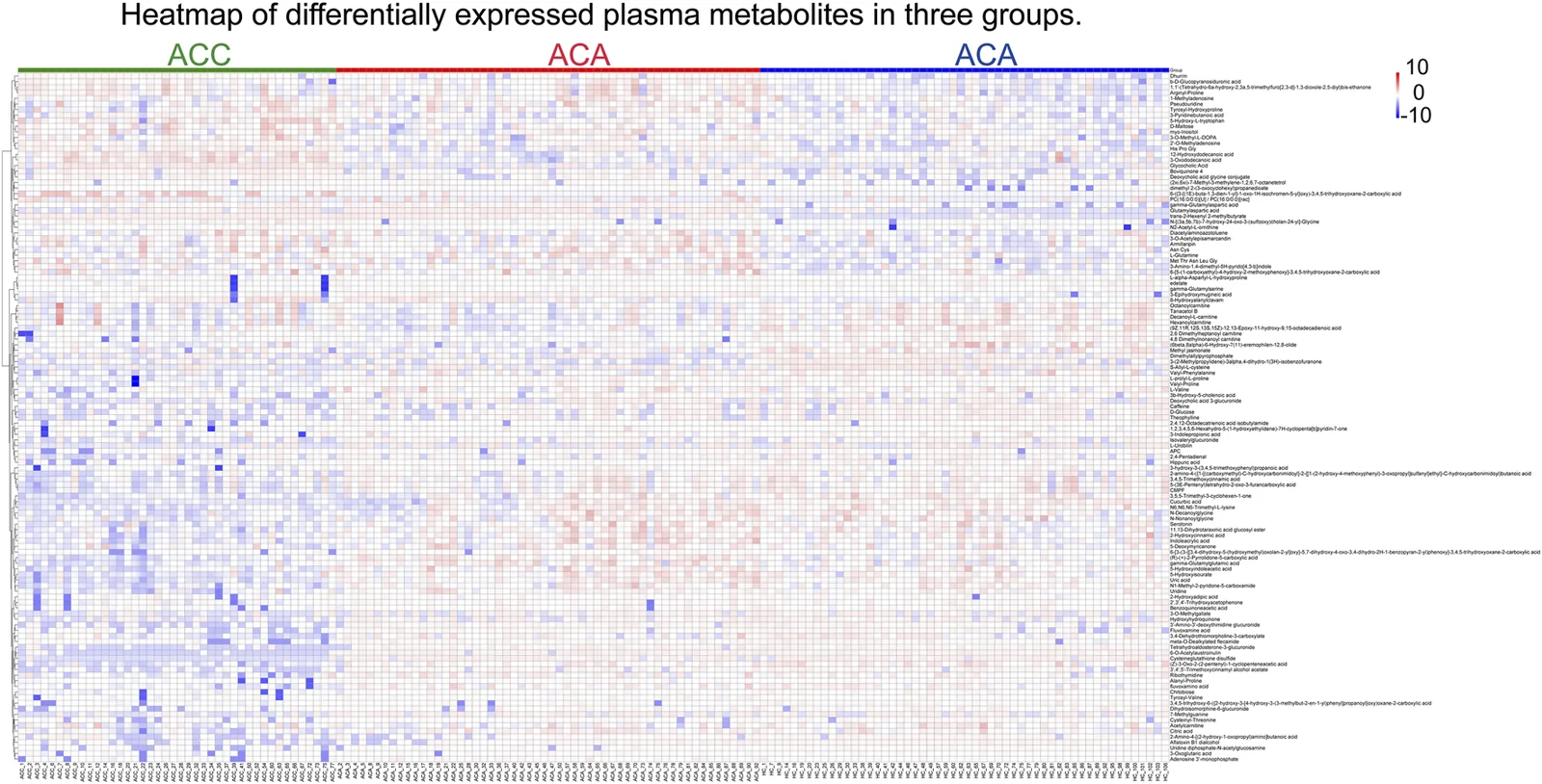
The quantitative analysis results of differentially expressed plasma metabolites in ACC, ACA, and HC groups are shown in the form of heat map.

**FIGURE 3:**
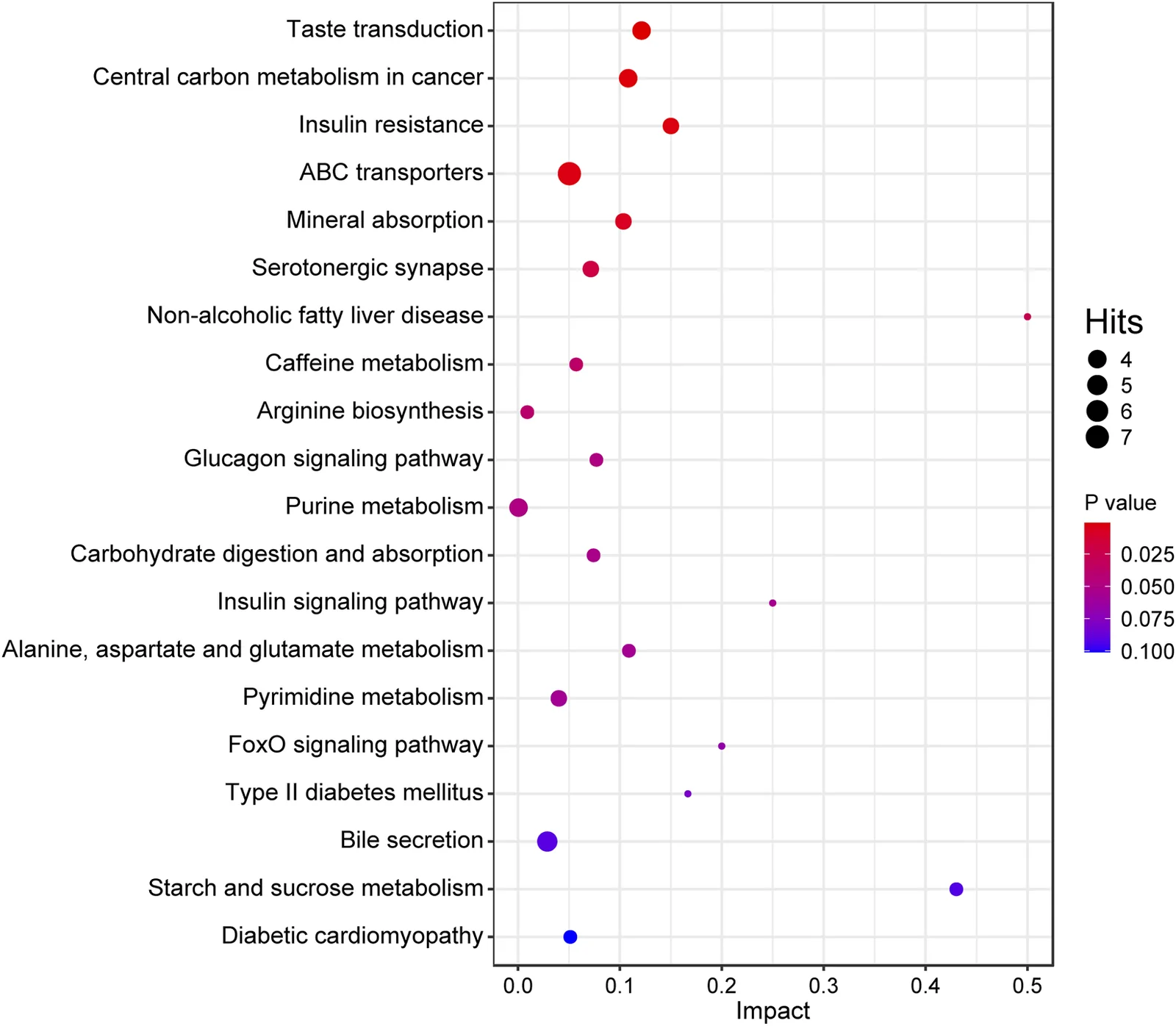
KEGG pathway analysis of 48 differentially expressed plasma metabolites in ACC, ACA, and HC groups.

In total, 488 metabolites were identified in the plasma of ACC, ACA, and healthy control groups. The differentially expressed plasma metabolites were selected based on the P-value, VIP, and FDR. Metabolites with *P* < 0.5, FDR < 0.1, and VIP > 1 were selected, and 123 metabolites were differentially expressed in the three groups (Figure 2), which demonstrated the specific alterations of plasma metabolites in the progression of ACC. Among those differentially expressed metabolites, 48 metabolites with KEGG ID were collected, and KEGG analysis was performed to explore the dysregulated metabolic pathway in the progression of ACC. As shown in Figure 3, 48 significantly high-expressed metabolites were significantly enriched in 11 pathways, namely, taste transduction, central carbon metabolism in cancer, insulin resistance, ABC transporters, mineral absorption, serotonergic synapse, non-alcoholic fatty liver disease, caffeine metabolism, arginine biosynthesis, glucagon signaling pathway, and purine metabolism. The results revealed that those dysregulated metabolites might be involved in multiple signaling pathways to affect the progression of ACC.

### 3.3 Diagnostic value analysis of the upregulated metabolites in the ACC group

**FIGURE 4:**
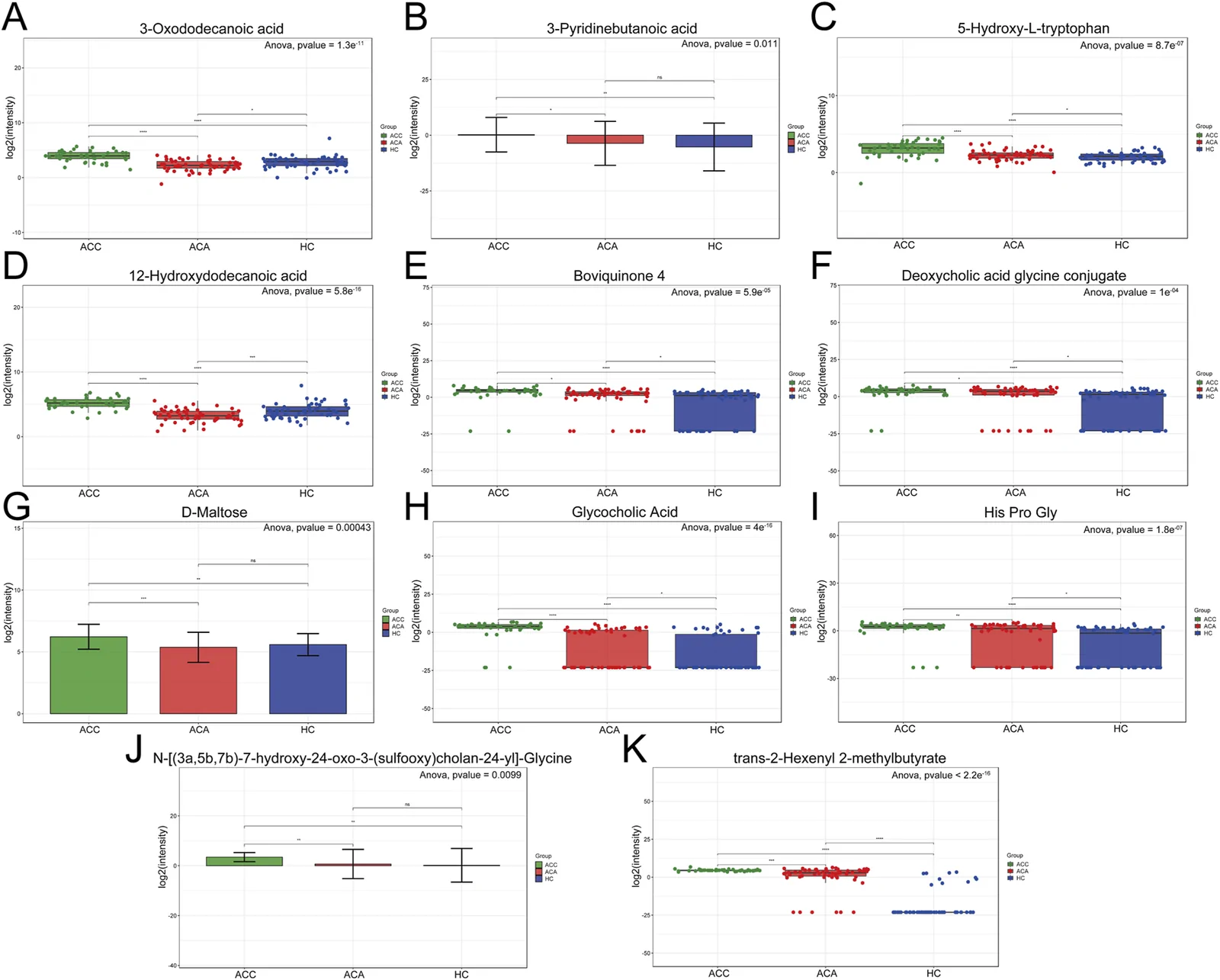
The relative concentration of 11 plasma metabolites in ACC, ACA, and healthy control groups, namely, 3-oxododecanoic acid **(A)**, 3-pyridinebutanoic acid **(B)**, 5-hydroxy-L-tryptophan **(C)**, 12-hydroxydodecanoic acid **(D)**, boviquinone 4 **(E)**, deoxycholic acid glycine conjugate **(F)**, D-maltose **(G)**, glycocholic acid **(H)**, His-Pro-Gly **(I)**, N-[(3a,5b,7b)-7-hydroxy-24-oxo-3-(sulfooxy) cholan-24-yl]-Glycine **(J)**, and trans-2-hexenyl-2-methylbutyrate **(K)**. *P < 0.05, **P < 0.01, ***P < 0.001, and ****P < 0.0001 vs. ACC group.

**FIGURE 5:**
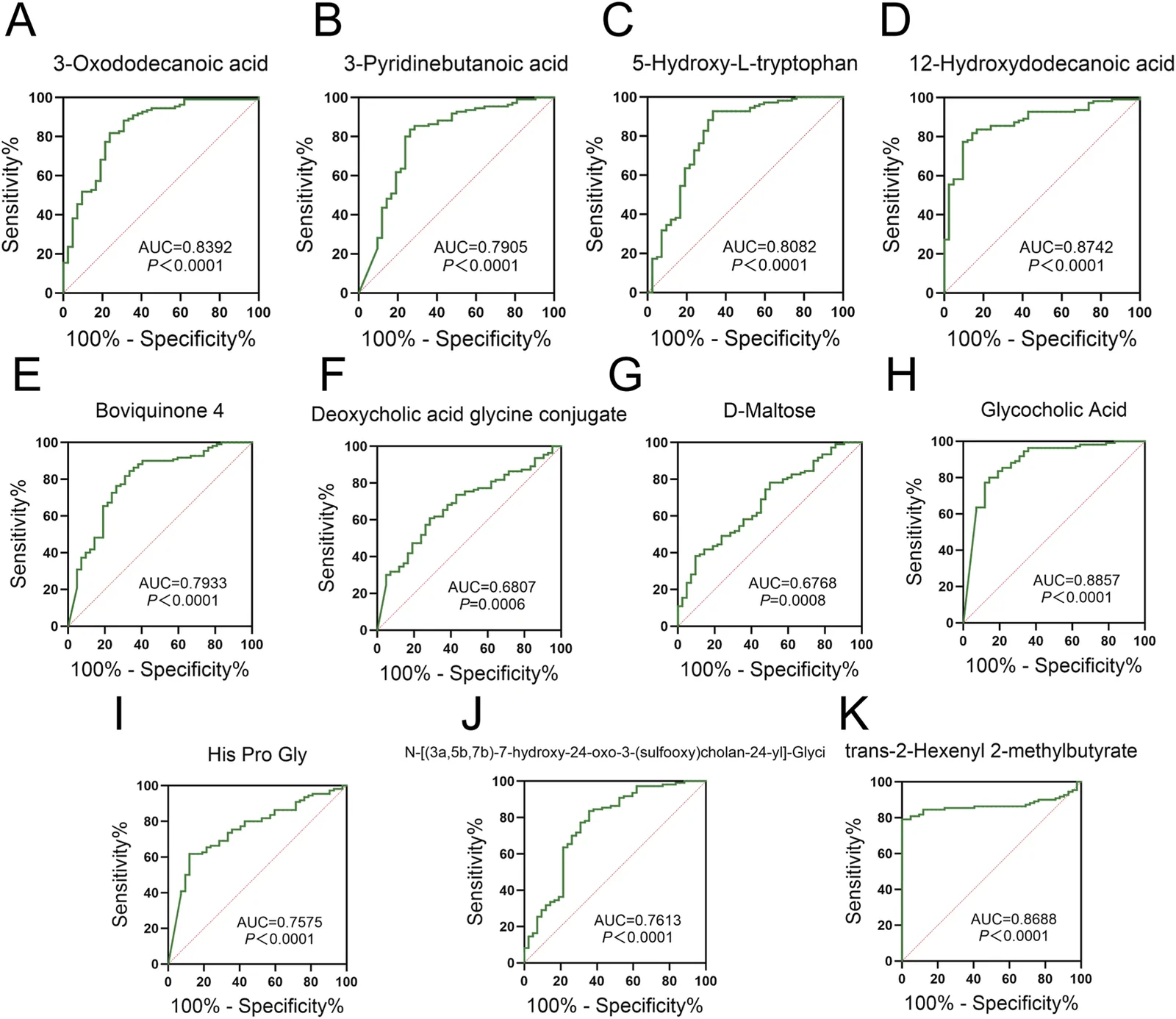
The ROC curves of 11 plasma metabolites for the diagnosis of ACC patients, namely, 3-oxododecanoic acid **(A)**, 3-pyridinebutanoic acid **(B)**, 5-hydroxy-L-tryptophan **(C)**, 12-hydroxydodecanoic acid **(D)**, boviquinone 4 **(E)**, deoxycholic acid glycine conjugate **(F)**, D-maltose **(G)**, glycocholic acid **(H)**, His-Pro-Gly **(I)**, N-[(3a,5b,7b)-7-hydroxy-24-oxo-3-(sulfooxy) cholan-24-yl]-Glycine **(J)**, and trans-2-hexenyl-2-methylbutyrate **(K)**.

To further explore plasma metabolites as potential diagnostic markers for ACC, plasma metabolites significantly enriched in ACC groups were selected, and the relative concentrations were quantified for comparison. As shown in Figure 4, a total of 11 metabolites were significantly upregulated in the ACC group compared with the ACA and healthy control groups, namely, 3-oxododecanoic acid, 3-pyridinebutanoic acid, 5-hydroxy-L-tryptophan, 12-hydroxydodecanoic acid, boviquinone 4, deoxycholic acid glycine conjugate, D-maltose, glycocholic acid, His-Pro-Gly, N-[(3a,5b,7b)-7-hydroxy-24-oxo-3-(sulfooxy)cholan-24-yl]-glycine, and trans-2-hexenyl-2-methylbutyrate. Among them, D-maltose had the highest plasma concentration. To further evaluate the diagnostic value of these upregulated metabolites in ACC group, the ROC analysis was performed to analyze the diagnostic accuracy of the abovementioned 11 metabolites in discriminating ACC from ACA and HC (Figure 5). Five metabolites had ROC curves with AUCs >0.8, namely, glycocholic acid, 12-hydroxydodecanoic acid, trans-2-hexenyl-2-methylbutyrate, 3-oxododecanoic acid, and 5-hydroxy-L-tryptophan, demonstrating the adequate prognosis value of those five plasma metabolites to discriminate ACC from ACA and HC. Among them, glycocholic acid, one of the primary conjugated bile acids, ranked first, with the highest AUC of 0.8857 (*P* < 0.0001), thus displaying the greatest potential for diagnosing ACC (Watling et al., 2025). To further evaluate whether glycocholic acid can predict the pathological stage of ACC patients, correlation analysis was performed between the level of plasma glycocholic acid and the ENSAT stage in the ACC group. However, no significant correlation was observed between plasma glycocholic acid levels and the ENSAT stage in the ACC group (Supplementary Figure S2). As an important bile acid, glycocholic acid binds to bile acid receptors to exert its biological functions. TGR5, characterized as a G-protein-coupled bile acid receptor, has been widely reported as double sword with tumor-suppressing and oncogenic functions (Qi et al., 2022). However, its role in ACC remains unclear. Considering the higher plasma level of glycocholic acid in ACC, the expression of TGR5 was explored in ACC. Surprisingly, the expression of TGR5 was downregulated in ACC (Supplementary Figure S3), but the underlying molecular mechanism needs further exploration.

### 3.4 The metabolic diagnostic model accurately predicts the diagnosis of ACC patients

**FIGURE 6:**
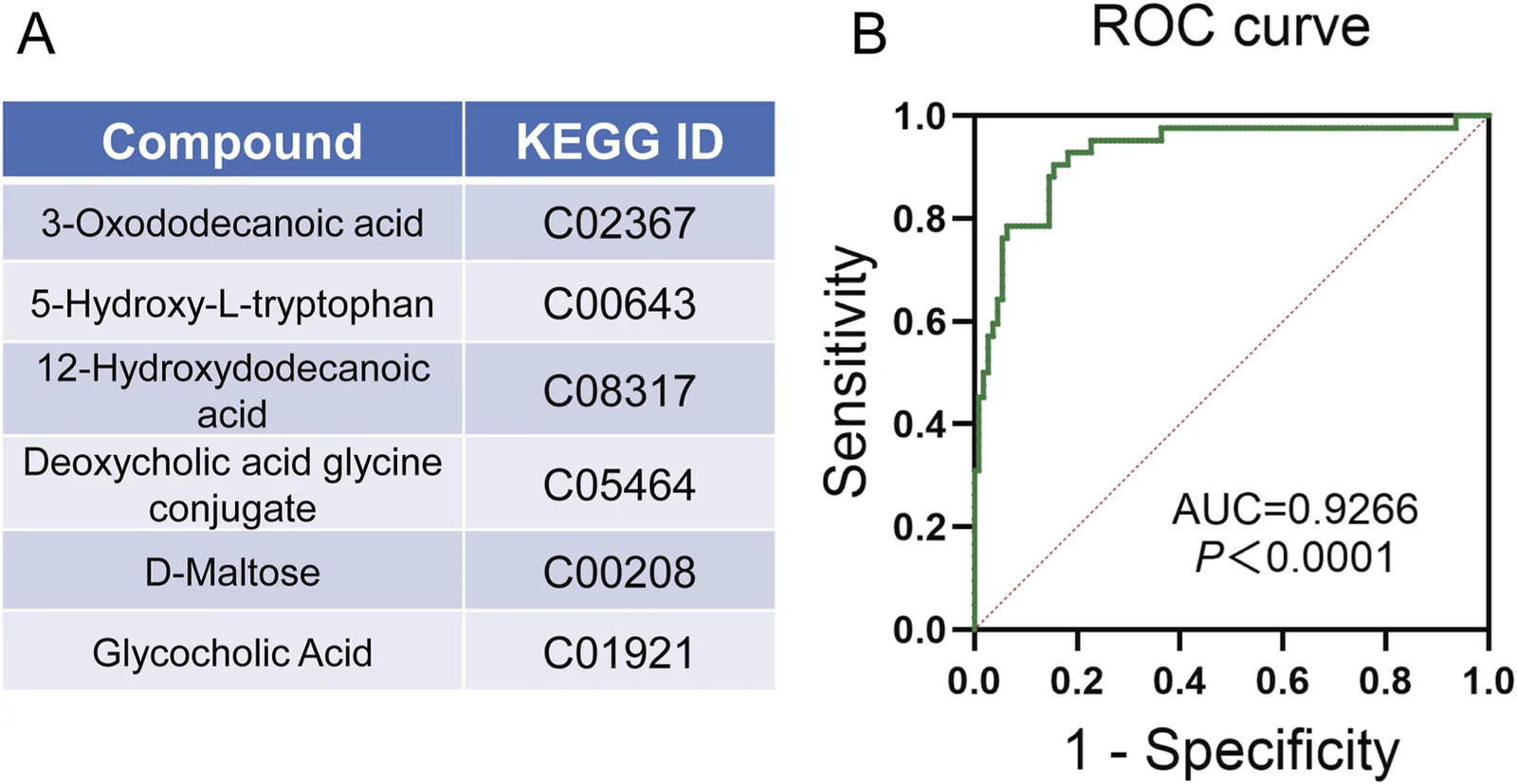
Multiple logistic regression was performed to evaluate the accuracy of the metabolic diagnostic model in discriminating ACC patients from ACA patients and healthy controls. **(A)** Six plasma metabolites with known KEGG ID. **(B)** The ROC curve of the predicted model.

Based on the above results, 11 plasma metabolites have been identified as significantly elevated, and their diagnostic values have been validated. To further construct a metabolic diagnostic model with more accuracy in predicting the diagnosis of ACC patients, six metabolites with known KEGG ID were selected to consist the metabolic diagnostic model. In addition, multiple logistic regression was performed to evaluate the ROC curve of the model (Figure 6A). As shown in Figure 6B, the metabolic diagnostic model consisting of the plasma concentration of 3-oxododecanoic acid, 5-hydroxy-L-tryptophan, 12-hydroxydodecanoic acid, deoxycholic acid glycine conjugate, D-maltose, and glycocholic acid realized a higher accuracy in discriminating ACC patients from ACA patients and healthy controls, with an AUC of 0.9266 of the ROC curve (*P* < 0.0001), indicating that the presenting metabolic diagnostic model accurately predicted the diagnosis of ACC patients. To assess the robustness and generalizability of the model, 10-fold stratified cross-validation was performed (Supplementary Figure S4). The mean AUC across the 10 folds was 0.912 ± 0.086 (95% CI: 0.850–0.974), confirming that the model maintained strong and consistent discriminative performance across independent test partitions. Collectively, these findings demonstrated that the six-metabolite panel constituted a potential biomarker signature for ACC diagnosis.

## 4 Discussion

In the present study, plasma untargeted metabolomics was performed to identify the potential diagnostic metabolites for discriminating ACC patients from ACA patients and healthy controls. Differential plasma metabolites were identified across three groups, and the KEGG pathway was enriched based on the differential plasma metabolites. Among the upregulated plasma metabolites in ACC patients, glycocholic acid was ranked first, with the highest diagnostic accuracy in the diagnosis of ACC patients. Moreover, a metabolic diagnostic model containing 3-oxododecanoic acid, 5-hydroxy-L-tryptophan, 12-hydroxydodecanoic acid, deoxycholic acid glycine conjugate, D-maltose, and glycocholic acid was constructed and accurately predicted ACC, with an AUC of 0.9266. Our research not only revealed the dysregulation of plasma metabolites during ACC progression but also identified the potential plasma metabolites for discriminating ACC patients from ACA patients and healthy controls.

As an aggressive and rare neoplasm, ACC was associated with a complex but poor prognosis with limited effective treatment regimens (Crona and Beuschlein, 2019; Cremaschi et al., 2022). Complete surgical resection is the fundamental treatment option for primary ACC (Russell, 2024). However, the limitation in the diagnosis of ACC versus benign ACA hinders treatment options (Imperiale et al., 2013). Some studies focused on the urine steroid metabolomics as a promising tool in the diagnosis of ACC since the innate dysregulated steroidogenic pattern (Arlt et al., 2011; Mizdrak et al., 2021; Bancos et al., 2020). Chortis et al. (2020) evaluated the performance of urine steroid metabolomics to detect postoperative recurrence in ACC and found the 11-deoxycortisol metabolite THS with the highest predictive potential. Vogg et al. (2025) proposed enzymatically deconjugated steroids as diagnostic biomarkers for adrenal tumors and discovered several urinary steroid conjugates with great accuracy to discriminate ACC from ACA. Schweitzer et al. (2019) investigated plasma steroid hormone profiling using LC–MS/MS for the diagnosis of ACC from ACA, but healthy controls were not included in this study. However, no relevant study has performed plasma metabolomics to assess the whole plasma metabolites to identify diagnostic biomarkers in plasma for ACC. To the best of our knowledge, we first performed plasma metabolomics for ACC patients, ACA patients, and healthy controls and identified glycocholic acid as the potential diagnostic biomarker for ACC, emphasizing plasma metabolomics as a promising tool for the detection of ACC.

As a secondary bile acid, glycocholic acid plays fundamental roles in the emulsification and digestion of fat (Li et al., 2016). Glycocholic acid is also involved in the development of several diseases, including cancer. It has been reported that glycocholic acid can aggravate liver fibrosis by promoting the expression of connective tissue growth factor (Yuan et al., 2023). Glycocholic acid has also been identified as an upregulated metabolite in urine samples of patients with liver cancer (Zhang et al., 2013). In cholangiocarcinoma (CCA), glycocholic acid was validated as a potential metabolic biomarker for the specific detection of CCA (Song et al., 2018). So far, no relevant study has explored the changes in glyocoholic acid in ACC, and we first reported that plasma glyocoholic acid could serve as a promising diagnostic biomarker to discriminate ACC from ACA and healthy controls. Moreover, it has been reported that serum bile acids may serve as risk factors in cancer, and elevated serum glycocholic acid promotes the expression of PD-L1 to impede CD8^+^ T cells-mediated anti-tumor immunity to promote colorectal cancer progression (Zhao et al., 2026). In parallel, our discoveries highlighted the potential significance of glycocholic acid in the progression of ACC. Moreover, we also found the bile acid receptor TGR5 was significantly downregulated in ACC, but the precise mechanism was still unclear. Our discovery not only highlights the potential role of serum glycocholic acid and its receptor in ACC progression but also reveals a novel dysregulated metabolic pathway that can be served as a potential therapeutic drug target for ACC. However, there was no relevant study revealing the function and mechanism of glycocholic acid in ACC. In light of elevated serum glycocholic acid in ACC, experimental validation should be conducted to explore the specific role for glycocholic acid in the progression of ACC in the future. In clinical settings, immunohistochemistry is still essential for the diagnosis and prognostic evaluation of ACC. A series of immunohistochemical markers are involved in the diagnosis and grading of ACC, including Ki67, SF1, and IGF2 (Vocino et al., 2026). Compared with traditional ACC diagnostic methods, plasma metabolomics is promising for complementing and addressing the deficiencies of immunohistochemistry, due to its non-invasive and convenient sampling method and high-sensitivity detection. Moreover, it is worthwhile to further explore the correlation analyses between bioactive plasma metabolites and key clinicopathological parameters in the diagnosis of ACC. The rapid and striking development of artificial intelligence can also be used to develop a precise prediction model by integrating the differentially expressed plasma metabolites and immunohistochemical markers, thus further facilitating the clinical translation in ACC precision diagnosis (Sahoo et al., 2025). Mitotane remains the cornerstone of ACC medical management, but its efficacy varies widely among ACC patients (Corso et al., 2021). Thus, six plasma metabolites hold promise not only as a non-invasive diagnostic tool but also as functional biomarkers for patient stratification to predicting the response of mitotane in ACC patients. Furthermore, in terms of evolving clinical trials, the present metabolite signature could serve as an enrichment criterion to match specific ACC cohorts with matched metabolic or molecular targeted therapies, moving toward precision oncology for this rare malignancy. Altogether, our results highlighted the significant roles of specific plasma metabolites in the progression of ACC and demonstrated the latent potential of plasma metabolites as biomarkers for early non-invasive diagnosis of ACC. However, due to the lack of robust external validation, our finding remains preliminary and exploratory, and the constructed model requires further confirmation through independent multi-center validation with larger sample sizes.

However, several limitations of this study should be acknowledged. First, although the study samples were derived from a consecutive clinical case database, cases with incomplete clinical or laboratory data were excluded from the final analysis. This exclusion may have introduced selection bias as the missing data might not have been completely random. Second, the modest sample size of the ACC group (n = 42) increases the risk of model overfitting. To address this, we implemented 10-fold cross-validation; nevertheless, internal cross-validation cannot substitute for independent external validation. Therefore, larger prospective cohorts are warranted to confirm our preliminary findings in future investigations. Third, owing to the retrospective design and the incomplete documentation of clinical records in the historical database, we were unable to perform correlation analyses between the identified metabolite biomarkers and important clinicopathological parameters, including Ki-67 proliferation index, tumor size, and hormonal functional status. The lack of these correlations limits our ability to fully evaluate whether the identified metabolites are associated with tumor aggressiveness or endocrine activity. Future prospective studies with systematically and uniformly collected clinical and pathological data are warranted to validate our findings and to further explore the relationships between plasma metabolomic signatures and key tumor characteristics. In addition, although glycocholic acid ranked as the most promising plasma metabolite for the diagnosis of ACC, it cannot accurately predict the pathological stage of ACC (Supplementary Figure S2). In the future, function validation experiments and molecular mechanism studies will be performed to further verify the role of glycocholic acid in the development and progression of ACC.

Altogether, we proposed plasma metabolomics as a promising tool for identifying potential diagnostic biomarkers in plasma metabolites. Glycocholic acid was first identified as a potential biomarker to distinguish ACC from ACA and healthy controls. Moreover, a reliable diagnostic signature for the diagnosis of ACC was constructed and realized high accuracy, consisting of the top six differentially expressed plasma metabolites with known KEGG ID, namely, 3-oxododecanoic acid, 5-hydroxy-L-tryptophan, 12-hydroxydodecanoic acid, deoxycholic acid glycine conjugate, D-maltose, and glycocholic acid. This model distinguished ACC from ACA and healthy controls with an AUC of 0.9266, demonstrating that plasma metabolomics can serve as a promising tool for the early detection of ACC.

## Data Availability

The raw data supporting the conclusions of this article will be made available by the authors, without undue reservation.

## Supplementary material

SUPPLEMENTARY FIGURE S1 QC evaluation of the metabolomics dataset. **(A)** PCA score plot including biological samples and retained QC injections after preprocessing. **(B)** Total quantified feature abundance across QC injections; retained QC injections showed a CV of 3.82%. **(C)** Injection-level filtering based on missing/nonpositive feature rate. QC injections with missing/nonpositive rate >25% were excluded before downstream QC evaluation.

SUPPLEMENTARY FIGURE S2 The content of glycocholic acid in different ENSAT stage in ACC group.

SUPPLEMENTARY FIGURE S3 The mRNA expression of TGR5 in ACC. |Log2FC| Cutoff: 0.5, p-value Cutoff: 0.05.

SUPPLEMENTARY FIGURE S4 10-fold cross-validation was performed as internal validation to assess model stability of multiple logistic regression.
