# Supplementary material for: Plasma untargeted metabolomics reveals promising diagnostic metabolites for adrenocortical carcinoma
Source: Front Pharmacol. 2026 Aug 21;17:1817514. doi: 10.3389/fphar.2026.1817514 (PMC13541528; doi:10.3389/fphar.2026.1817514)
Supplement: Supplementary file 1 [file DataSheet1.docx]

**Supplementary materials**

**Plasma untargeted metabolomics reveals promising diagnostic metabolites for adrenocortical carcinoma**

Yihui Yang^1,2,^ ^†^, Shuping Shi^1,2,^ ^†^,Jianqiang Wu^3^, Tingting Xu^1,2^, Junmei Shang^1,2^, Jiaxin Yu^1,2^, Bo Zhang^1,2*^, Xin Liu^1,2*^

^1^Department of Pharmacy, Peking Union Medical College Hospital, Chinese Academy of Medical Sciences and Peking Union Medical College, Beijing, China, ^2^State Key Laboratory of Complex Severe and Rare Diseases, Peking Union Medical College Hospital, Beijing, China,^3^ Center for Biomarker Discovery and Validation, National Infrastructure for Translational Medicine, Institute of Clinical Medicine, Peking Union Medical College Hospital, Chinese Academy of Medical Sciences and Peking Union Medical College, Beijing 100730, China

†These authors have contributed equally to the work.

*Correspondence to Xin Liu (twinsyuanxin@126.com) and Bo Zhang (zhangbo@pumch.cn)

**Supplementary figures**

**Supplementary figure 1. QC evaluation of the metabolomics dataset.** (A) PCA score plot including biological samples and retained QC injections after preprocessing. (B) Total quantified feature abundance across QC injections; retained QC injections showed a CV of 3.82%. (C) Injection-level filtering based on missing/nonpositive feature rate. QC injections with missing/nonpositive rate >25% were excluded before downstream QC evaluation.

**Supplementary figure 2. The content of glycocholic acid in different ENSAT stage in ACC group.**

**Supplementary figure 3. The mRNA expression of TGR5 in ACC. |Log2FC| Cutoff: 0.5, p-value Cutoff:0.05.**

**Supplementary figure 4. 10-fold cross-validation was performed as internal validation to assess model stability of multiple logistic regression.**
